# Increased myocardial extracellular volume in active idiopathic systemic capillary leak syndrome

**DOI:** 10.1186/s12968-015-0181-6

**Published:** 2015-08-27

**Authors:** Andrew Ertel, Drew Pratt, Peter Kellman, Steve Leung, Patricia Bandettini, Lauren M. Long, Michael Young, Celeste Nelson, Andrew E. Arai, Kirk M. Druey

**Affiliations:** National Heart, Lung, and Blood Institute, National Institutes of Health, Bethesda, MD USA; Medstar Washington Hospital Center, Washington, DC USA; Laboratory of Pathology, National Cancer Institute, National Institutes of Health, Bethesda, MD USA; University of Kentucky, Lexington, KY USA; National Institute of Allergy and Infectious Diseases, National Institutes of Health, 50 South Drive Room 4154, Bethesda, MD 20892-8305 USA

**Keywords:** Systemic capillary leak syndrome, Mycocardial edema, Cardiovascular magnetic resonance

## Abstract

**Background:**

The Systemic Capillary Leak Syndrome (SCLS) is a rare disorder of unknown etiology presenting as recurrent episodes of shock and peripheral edema due to leakage of fluid into soft tissues. Insights into SCLS pathogenesis are few due to the scarcity of cases, and the etiology of vascular barrier disruption in SCLS is unknown. Recent advances in cardiovascular magnetic resonance (CMR) allow for the quantitative assessment of the myocardial extracellular volume (ECV), which can be increased in conditions causing myocardial edema. We hypothesized that measurement of myocardial ECV may detect myocardial vascular leak in patients with SCLS.

**Methods:**

Fifty-six subjects underwent a standard CMR examination at the NIH Clinical Center from 2009 until 2014: 20 patients with acute intermittent SCLS, six subjects with chronic SCLS, and 30 unaffected controls. Standard volumetric measurements; late gadolinium enhancement imaging and pre- and post-contrast T1 mapping were performed. ECV was calculated by calibration of pre- and post-contrast T1 values with blood hematocrit.

**Results:**

Demographics and cardiac parameters were similar in both groups. There was no significant valvular disorder in either group. Subjects with chronic SCLS had higher pre-contrast myocardial T1 compared to healthy controls (T1: 1027 ± 44 *v*. 971 ± 41, respectively; *p* = 0.03) and higher myocardial ECV than patients with acute intermittent SCLS or controls: 33.8 ± 4.6, 26.9 ± 2.6, 26 ± 2.4, respectively; *p* = 0.007 *v*. acute intermittent; *P* = 0.0005 *v*. controls). When patients with chronic disease were analyzed together with five patients with acute intermittent disease who had just experienced an acute SCLS flare, ECV values were significantly higher than in subjects with acute intermittent SCLS in remission or age-matched controls and (31.2 ± 4.6 %, 26.5 ± 2.7 %, 26 ± 2.4 %, respectively; *p* = 0.01 *v*. remission, *p* = 0.001 *v*. controls). By contrast, T1 values did not distinguish these three subgroups (1008 ± 40, 978 ± 40, 971 ± 41, respectively, *p* = 0.2, active *v*. remission; *p* = 0.06 active *v*. controls). Abundant myocardial edema without evidence of acute inflammation was detected in cardiac tissue postmortem in one patient.

**Conclusions:**

Patients with active SCLS have significantly higher myocardial ECV than age-matched controls or SCLS patients in remission, which correlated with histopathological findings in one patient.

## Background

Patients with SCLS (also known as Clarkson disease) experience transient but recurring episodes of hypotensive shock followed by generalized edema of the face, trunk, and extremities [1]. Although vascular leakage in SCLS may result from transient, reversible endothelial barrier dysfunction induced by humoral mediators [2, 3], the identity of the pathogenic factors and their vascular target(s) remain unknown. Triggers for SCLS flares are not apparent although episodes may be preceded by viral infections, suggesting a role for inflammation in the induction of acute vascular leak [4]. Because patients with SCLS feel well between episodes and appear healthy, it is uncertain whether they have ongoing (subclinical) vascular barrier dysfunction. The fluid shifts associated with acute SCLS can lead to cardiopulmonary and renal failure and compartment syndromes of the extremities, resulting in chronic sensorimotor defects and loss of limb(s) [1, 5]. The 5-year mortality due to SCLS in the absence of prophylactic treatment has been estimated to be as high as 80 % [4]. However, a longitudinal follow up study of patients treated prophylactically with high dose intravenous immunoglobulin (IVIG) on a monthly basis revealed a significant reduction in the frequency of SCLS flares compared to pre-IVIG frequencies [6].

A significant obstacle to research into the pathogenesis of SCLS is the extreme rarity of the disease. Fewer than 300 cases have been reported since its original description in 1960, and current worldwide prevalence is estimated to be ~100 cases with a confirmed diagnosis [1]. Because there are no biomarkers or diagnostic modalities that are specific to SCLS, the diagnosis is made on the basis of a triad of clinical signs: hypotension, elevated hematocrit, and hypoalbuminemia, which are thought to result from plasma extravasation into tissues. A subset of patients with SCLS experiences chronic, noncyclical edema and hypoalbuminemia, which may or may not be accompanied by periodic hypotensive episodes. It is uncertain whether these patients have Clarkson disease or a distinct disorder.

Although we hypothesize that the primary vascular defect in SCLS resides in peripheral tissues, several case reports have provided evidence of primary myocardial involvement [7, 8]. A young man with SCLS and hypotensive shock resistant to fluid resuscitation and inotropic support was described recently, which required extracorporeal life support during the leak phase [9]. Echocardiography revealed global left ventricular systolic dysfunction during the leak phase, and myocardial biopsy demonstrated extensive interstitial edema without inflammatory infiltrates or myocyte necrosis. Systolic function returned to normal upon resolution of the episode.

Recently, techniques have been described to quantify the extracellular volume fraction (ECV) of the myocardium with cardiovascular magnetic resonance (CMR) [10, 11]. Myocardial ECV is increased in conditions in which myocardial tissue is replaced by fibrosis including chronic myocardial infarction; in conditions causing interstitial infiltration, such as cardiac amyloidosis; and in conditions causing interstitial edema, including acute myocardial infarction and myocarditis [12]. We hypothesized that ECV measurements would enable detection of myocardial edema in subjects with active SCLS. Indeed, in a preliminary study, two subjects with chronic SCLS had significantly higher ECV values than did a group of 62 healthy controls [13]. In the present study, we quantified myocardial ECV in an expanded cohort of 26 subjects with acute, intermittent and chronic forms of SCLS and 30 age-matched controls without overt cardiovascular disease.

## Methods

### Patients

Patients were classified with acute intermittent systemic capillary leak syndrome on the basis of having one or more episodes associated with the diagnostic clinical triad of 1) hypotension, 2) elevated hematocrit, and 3) hypoalbuminemia, using established criteria [4]. Patients were identified with chronic systemic capillary leak syndrome on the basis of having persistent unremitting peripheral edema and hypoalbuminemia in the absence of any other underlying causes including primary cardiac, renal, endocrine, or hepatic disease. Control subjects were defined as individuals undergoing clinically-indicated contrast-enhanced CMR scans for suspected cardiovascular disease, whose studies demonstrated normal ventricular size and systolic function, no significant valvular abnormalities, normal vasodilator perfusion imaging (for patients in whom stress testing was performed) and an absence of myocardial enhancement on late gadolinium enhancement imaging.

### Cardiovascular magnetic resonance

Subjects with documented SCLS and control patients underwent ECV imaging prospectively between 2009 and 2014. Hematocrit was measured from a venous blood sample obtained immediately prior to the CMR study. The ECV imaging protocol used in this study was approved by the local Institutional Review Boards of the National Heart, Lung and Blood Institute and Suburban Hospital (Bethesda, MD). All subjects gave written informed consent to participate. CMR was performed with a 1.5 T MRI scanner (Magnetom Avanto, Espree or Aera, Siemens Healthcare Sector, Erlanger, Germany) using a 30 or 32-channel coil. Steady-state free precession cine images (6 mm slice thickness, echo time 1.2 ms, flip angle 50°) were obtained in short axis view (stack from base to apex with 4 mm interslice gap) and 3 long axis views (4-chamber, 3-chamber, 2-chamber). T1 quantification was performed using a modified Look-Locker inversion-recovery (MOLLI) sequence [14] acquired in a mid-ventricular short axis and a 4-chamber long axis plane before and approximately 15 min after intravenous administration of a gadolinium-based contrast agent (0.15 mmol/kg of either gadopentate dimeglumine [Magnevist^®^) or gadobutrol (Gadavist^®^) (Bayer Healthcare Pharmaceuticals, Wayne, NJ, USA)]. Typical MOLLI imaging parameters were: non-selective inversion pulse, steady state free precession single shot readout with 35° excitation flip angle, field of view 360 × 270 mm^2^, slice thickness 6 mm, minimum inversion 110 ms, inversion time increment 80 ms, matrix 256 × 144, voxel size 2.1 × 1.9 × 6.0 mm^3^, time to repetition/time to echo of 2.7/1.1, parallel imaging acceleration with a factor of 2. Both pre- and post-contrast T1 maps were acquired using the same imaging parameters. Late gadolinium enhancement images were obtained starting approximately 10 min post-gadolinium-administration using a gradient recalled echo phase-sensitive inversion recovery technique (slice thickness 8 mm, echo time: 3.3 ms, flip angle: 25°) [15]. Optimal inversion time was selected by review of synthetic magnitude inversion recovery images generated during a post-contrast MOLLI acquisition and adjusted as needed to null the myocardium.

### ECV quantification

Calculation of the ECV is based on comparison of the longitudinal relaxation time constant (T1) of myocardium before and after administration of a gadolinium-based contrast agent (GBCA), taking advantage of the properties of the GBCA, which cannot enter myocardial cells and is thus exclusively present in the extracellular space. The GBCA shortens the T1 of the myocardium; thus, any difference in T1 between pre and post-contrast imaging is related to the amount of the GBCA in the extracellular space.

**Fig. 1:**
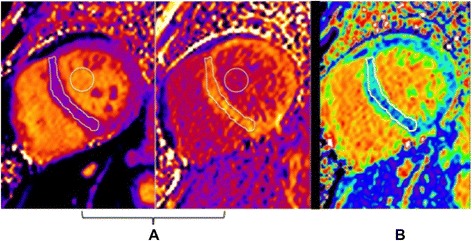
CMR findings in SCLS. **a** Pre-and post-contrast T1 maps in the mid-ventricular short axis of a patient with SCLS. Regions of interest drawn within the septum and blood pool are used to calculate ECV. **b** Mid-ventricular short axis ECV map in the same patient. The color scale for displaying ECV values was chosen so that green colors represent the mean ± 3 standard deviations of normal myocardium from age-matched controls

Myocardial ECV measurements were generated in one of two ways, both of which rely on T1 quantification techniques utilizing the MOLLI sequence as described in detail elsewhere [14]. Briefly, pre- and post-contrast T1 images obtained from the MOLLI sequence were motion-corrected and reformatted into a pixel map of T1 values. In our initial approach, average T1 values were quantified from regions of interest drawn manually within the mid-ventricular septum and blood pool on both pre-contrast and post-contrast T1 pixel maps (Fig. 1a). Because the change in myocardial relaxation rate ΔR1 (where R1 = 1/T1) between pre and post-contrast is directly proportional to the extracellular concentration of the GBCA, myocardial ECV can be estimated as follows (assuming contrast equilibrium between blood and myocardium) [10, 11].

$$ ECV=\left(1\hbox{-} hematocrit\right)\frac{\left(\frac{1}{T{1}_{myo\  post}}\hbox{---} \frac{1}{T{1}_{myo\ pre}}\right)}{\left(\frac{1}{T{1}_{blood\  post}}\hbox{---} \frac{1}{T{1}_{blood\ pre}}\right)} $$

The factor (1-hematocrit) represents the blood volume of distribution of gadolinium (blood ECV) and converts the equation from a partition coefficient calculation to the myocardial ECV. Recently, an automated method for producing a pixel-wise map of ECV has been described and validated, allowing direct measurement of myocardial ECV [12]. This method relies on co-registration of the pre- and post-contrast T1 pixel maps, correcting for respiratory motion due to insufficient breath-holding as well as patient movement between breath-holds. In this approach, measurement of ECV was obtained directly from regions of interest drawn manually within the mid-ventricular septum on the ECV pixel map.

### Histology

Representative samples from the left ventricular free wall, septum, and right ventricular free wall were fixed in 10 % neutral buffered formalin, embedded in paraffin and cut into 5 μm sections. Tissue sections were stained with hematoxylin and eosin and Masson’s trichrome. Immunohistochemical (IHC) staining for CD3 (2GV6, rabbit monoclonal antibody, Ventana Medical Systems, Tucson, Arizona, USA) and CD68 (KP-1, Ventana) was performed and detected by Basic-diaminobenzidine (DAB) using an automated immunostainer (BenchMark Ultra, Ventana). Whole slide imaging (WSI) was acquired using the Aperio digital imaging system (Aperio ScanScope XT scanner; Aperio Technologies, Vista, California, USA).

### Statistical analysis

The data are presented as mean ± s.e.m. unless otherwise noted. Differences between means were tested by the Mann-Whitney-U test or Kruskal-Wallis for non-parametric distributions. *P* values < 0.05 were considered significant.

## Results

### Clinical characteristics

We performed CMR in 26 subjects with SCLS and in 30 age-matched control subjects. Amongst the SCLS group, six patients had a history compatible with chronic SCLS. Although two of these subjects had a history of acute SCLS flares characterized by hypotension and hemoconcentration, they were included in the “chronic” subset owing to prominent and persistent edema in between episodes. One subject had atypical disease, with weekly episodes of hypotension but no peripheral edema that nonetheless fulfilled the diagnostic criteria for SCLS, and was therefore included in the analysis of the acute intermittent subgroup [3]. The remaining 19 subjects had prototypical acute intermittent SCLS as defined previously [1, 4]. All patients with acute intermittent SCLS did not have clinical manifestations of vascular leak (e.g. edema) at the time of CMR examination.

Baseline demographics are reported in Table 1. Parameters (age, sex etc.) did not differ significantly between the control and SCLS subgroups. At the time of the procedure, 46 % (11/26) of the patients with SCLS were being treated with theophylline and terbutaline while five subjects were receiving monthly prophylaxis with IVIG. Nine patients were not receiving any prophylactic therapy prior to undergoing CMR principally because the diagnosis of SCLS had not yet been confirmed.

**Table 1:** Clinical characteristics of patients undergoing CMR

	SCLS acute intermittent	SCLS chronic	Controls
Number	20	6	30
Age (y)^a^	51 (40–70)	44 (31–64)	52 (32–77)
Male/Female	13/20	1/6	14/30
Height (cm)^b^	172 (11)	162 (11)	170 (10)
Weight (kg)^b^	80 (4)	89 (19)	81 (19)
Body surface area (m^2^)^b^	2 (0.2)	2 (0.3)	2 (0.2)
Body mass index (kg/m^2^)^b^	27 (4)	33 (5)	28 (5)
Hematocrit^b^	42 (6)	39 (4)	40 (3)
Serum creatinine (mg/dL)^b^	0.9 (0.2)	0.9 (0.3)	1 (0.2)
Creatinine clearance (mL/min)^b,c^	105 (36)	129 (30)	93 (34)
Glomerular filtration rate (mL/min/1.73 m^2^)^b,d^	86 (5)	85 (38)	71 (15)

^a^median (range)

^b^mean (S.D.)

^c^Cockcroft-Gault equation

^d^Modification of Diet in Renal Disease (MDRD) Study equation

### CMR findings

Left ventricular (LV) parameters including end systolic and diastolic volumes, mass, and ejection fraction were similar in subjects with acute intermittent SCLS, chronic SCLS, and controls (Table 2). Quantitative LV volumetric parameters could not be obtained for one SCLS patient due to poor image quality. Late gadolinium enhancement imaging (LGE) was normal in all control patients. In the SCLS population, one patient had evidence of a subsegmental myocardial infarction in the apical inferior wall; one patient had non-diagnostic LGE imaging due to motion artifact; and three patients had nonspecific atypical patterns of LGE. Due to the small number of patients with LGE, a comparison of ECV measurements between SCLS patients with and without LGE was not performed.

**Table 2:** Cardiac parameters detected by CMR

	SCLS acute intermittent	SCLS chronic	Controls
Left Ventricular (LV) ejection fraction (%)^a^	62 (5)	66 (8)	64 (5)
LV end diastolic volume (mL)^a^	145 (36)	153 (23)	144 (33)
LV end systolic volume (mL)^a^	56 (17)	54 (19)	52 (16)
LV mass (g)^a^	103 (26)	88 (17)	90 (28)

^a^mean (S.D.)

### T1 mapping and extracellular volume measurements

**Fig. 2:**
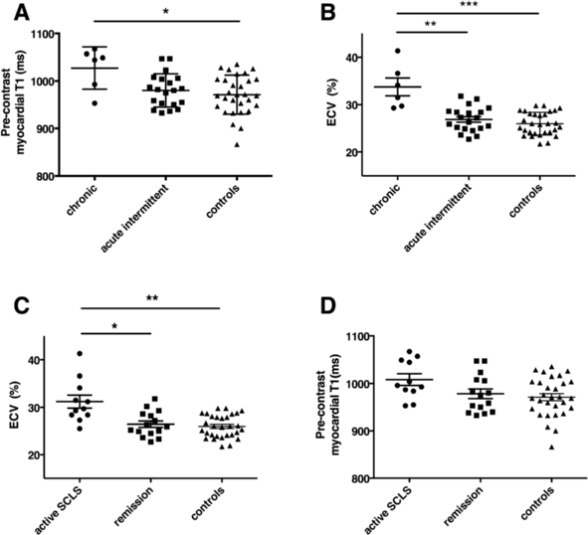
Quantitative analysis of pre-contrast T1 and myocardial ECV in SCLS and controls. **a**–**b** Pre-contrast myocardial T1 (**a**) or ECV (**b**) values were quantified by CMR in patients with chronic SCLS, acute intermittent SCLS, or controls. T1: *p = 0.03; ECV: **p = 0.007; ***p = 0.0005, Kruskall-Wallis. **c**–**d** ECV (**c**) or T1 (**d**) values in patients with active SCLS symptoms regardless of formal classification, patients with periodic disease in remission, or controls. *p = 0.01; **p = 0.001, Kruskall-Wallis

Pre-contrast T1 values did not differ significantly between patients with SCLS and healthy controls overall (991 ± 42 v. 971 ± 41, *p* = 0.15). In contrast, myocardial ECV measurements were significantly higher in the SCLS cohort than in the control group (28.5 ± 4.2 % vs. 26.0 ± 2.4 %; *P* = 0.02). ECV values for the patient group overall were nonetheless within the ± 2 standard deviation (s.d.) range established in our previous large-scale study of patients without known cardiac pathology (20.4–30.4 %) [13]. The highest ECV measurements were found in patients with noncyclical edema due to chronic SCLS, which primarily accounted for the statistical difference between SCLS subjects as a whole and controls. Accordingly, pre-contrast T1 values were higher in patients with chronic SCLS than in controls (1027 ± 44 *v*. 971 ± 41; *p* = 0.03 (Fig. 2a). ECV values in patients with chronic SCLS were also significantly higher than in the subset with acute intermittent SCLS or in the control group [33.8 ± 4.6 %, 26.9.0 ± 2.6 %, 26 ± 2.4 %, respectively; *p* = 0.007 *v*. acute intermittent; *p* = 0.0005 *v*. controls] (Fig. 2b). High myocardial ECV was not restricted to those subjects with chronic, non-cyclical edema, however. ECV values in the group of patients having what we termed “active” SCLS (which included both those with chronic edema and patients with acute intermittent disease, whose CMR was performed within a month of an acute flare) were significantly higher than in patients whose disease was in remission or healthy controls (31.2 ± 4.5 % *v*. 26.5 ± 2.7 v. 26 ± 2.4; *p* = 0.001 *v*. remission, *p* = 0.001 *v*. controls) (Fig. 2c). In contrast, pre-contrast T1 values did not differ significantly between these three groups (1008 ± 40, 978 ± 40, 971 ± 41, respectively) (Fig. 2d).

**Fig. 3:**
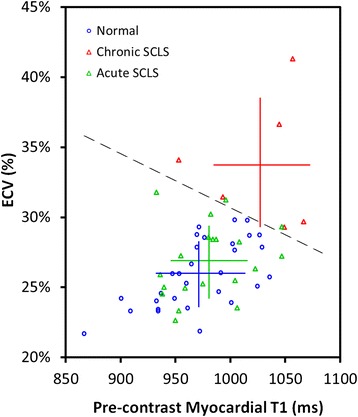
Relationship between pre-contrast myocardial T1 and myocardial ECV. A 2-dimensional analysis of T1 and ECV was performed. Linear discriminate analysis (dashed line) separates the chronic SCLS group from the combination of healthy controls and acute intermittent SCLS (*p* < 0.001)

We explored whether the combination of T1 and ECV could discriminate the different patient groups using a 2-dimensional plot. This analysis revealed a great amount of overlap between the paired T1-ECV points for healthy control subjects and those with acute intermittent SCLS (Fig. 3). Values for the chronic SCLS group are shifted up and to the right of both groups. The dashed line indicates that it is possible to separate the chronic SCLS group from normal in all cases in the 2-dimensional analysis, a degree of separation that is not achieved by T1 or ECV as individual measurements. That same line separates chronic SCLS from acute intermittent SCLS in remission for all but two of the acute intermittent SCLS patients. Linear discriminate analysis was able to separate the chronic SCLS group from the combination of healthy controls and acute intermittent SCLS (*p* < 0.001). ECV was a stronger predictor chronic SCLS than native T1 (standardized canonical discriminant function coefficients 0.95 *v*. 0.11).

### Histopathological analysis of myocardial edema

In order to further explore the etiology of increased myocardial ECV in SCLS, we evaluated postmortem cardiac tissue from the single patient in the cohort who died of SCLS during the study period for pathologic features of edema and/or fibrosis. This patient was a 54-year-old man who presented with hypotension (systolic blood pressures in the 80’s), haemoconcentration (Hgb > 20 g/dL), and hypoalbuminemia (<1.5 g/dL) after experiencing upper respiratory symptoms for several days. He had presented similarly 10 years prior to the current episode, at which time he developed severe edema leading to compartment syndromes of the arms and legs requiring fasciotomies. Subsequently, he had experienced only minor episodes of peripheral edema while receiving prophylaxis with theophylline. Recalcitrant hypotension requiring multiple vasopressors, compartment syndromes of the extremities requiring fasciotomies, and prolonged metabolic acidosis with multi-organ failure complicated his clinical course. The patient nearly exsanguinated from fasciotomy site bleeding due to disseminated intravascular coagulation and eventually developed ischemic necrosis of the right hand.

**Fig. 4:**
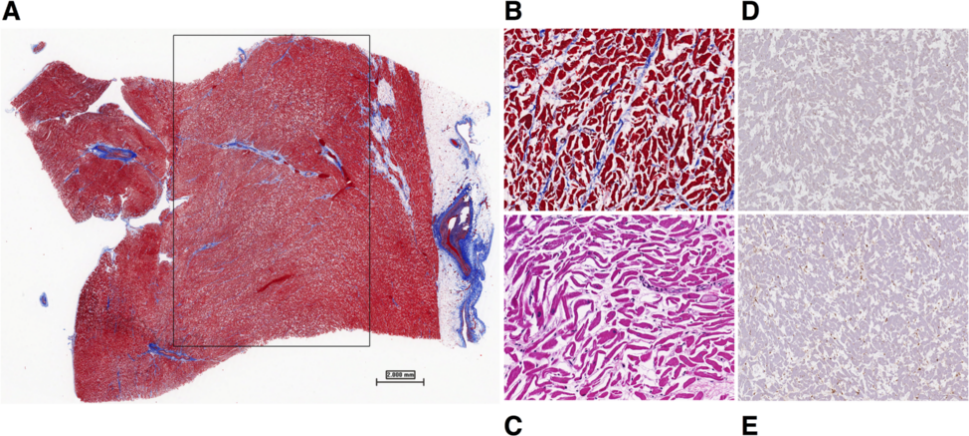
Postmortem histopathologic findings in a patient who died of SCLS. **a** Low power view of the left ventricle demonstrates diffuse myocardial edema, as highlighted in the enclosed area. (Masson’s trichrome histochemical stain, 0.6×) (**b**) Higher power view shows lace-like interstitial fibrosis between myofibers (Masson’s trichrome, 20×) (**c**) High power view of myocytes shows marked interstitial edema with an absence of myocyte necrosis (Hematoxylin and eosin, 20×) Immunohistochemical staining (IHC) for CD3 (d) and CD68 (**e**) show a paucity of inflammatory cells and the presence of scattered fibroblasts and few histiocytes. (8×)

Full-thickness sections of the left ventricle from this patient obtained postmortem demonstrated diffuse edema within the myocardial wall (Fig. 4a). There was lace-like fibrosis of the interstitial space in the myocardium, consisted with sustained and/or recurrent, intermittent episodes of myocardial edema (Fig. 4b). We did not detect appreciable myocyte necrosis (Fig. 4c) or lymphocytic infiltrates (Fig. 4d–e), features that would indicate a primary inflammatory myocarditis.

## Discussion

SCLS is a vastly understudied disorder of unknown etiology, with few effective therapies, and without specific biomarkers or diagnostic tests. We hypothesized that myocardial edema was present in patients with SCLS who have symptoms of active vascular leakage in peripheral tissues, and we evaluated the utility of myocardial ECV mapping in the diagnosis. In a cohort of 26 patients with SCLS, myocardial ECV was significantly higher than in a cohort of age-matched individuals without cardiovascular disease. Myocardial ECV in patients with the chronic form of SCLS and in patients with the acute form of SCLS who had had a recent episode (within 1 month) represented the highest values and largely accounted for the observed difference. In patients with acute SCLS in whom there was no recent episode, there was significant overlap in ECV measurements between patients and controls. This finding speaks to the dynamic, reversible nature of the vascular leak process in SCLS and the need for early imaging after an acute episode. In point of fact, inter-episode myocardial ECV was not elevated in our patient who succumbed to an acute SCLS episode, supporting the hypothesis that vascular barrier function is largely intact in subjects with SCLS in remission. Unfortunately, this patient was too hemodynamically unstable to perform ECV measurements during the acute exacerbation. It is also possible that subtle defects in vascular barrier function in patients with subclinical vascular leak may not be discernable by ECV mapping relative to the ± 2 s.d. range of normal myocardial ECV values (20.4–30.4 %) [12].

Myocardial T1 mapping and ECV mapping have emerged as promising new techniques, which can complement conventional cardiac MRI imaging sequences in cases of homogeneously diffuse myocardial disease states that affect the myocardial extracellular space. Previous cardiac MRI techniques have been well validated in the assessment of focal regions of myocardial fibrosis and edema. Late gadolinium enhancement imaging is considered the gold standard for imaging of focal myocardial fibrosis [16]. T2-weighted imaging techniques have been well validated in the assessment of focal myocardial edema in acute myocardial infarction and acute myocarditis [17]. Both of these techniques, however, rely on signal intensity differences between normal and abnormal myocardium to define pathology, and are not optimized for the assessment of diffuse myocardial abnormalities.

T1 and ECV mapping techniques can quantitatively characterize diffuse myocardial abnormalities not clinically apparent on LGE or T2-weighted images in a variety of disease states. T1 and ECV mapping can accurately detect diffuse increases in the interstitial space due to infiltrative cardiomyopathies. Recent studies have demonstrated a role for both noncontrast T1 mapping and ECV mapping in the accurate diagnosis of cardiac amyloidosis [18, 19]. T1 mapping/ECV mapping can also quantitatively characterize diffuse fibrosis associated with myocarditis [13], hypertrophic cardiomyopathy [20], anthracycline cardiotoxicity [21], and non-ischemic dilated cardiomyopathies [13]. Notably, increased ECV was also found in patients with systemic sclerosis, a disorder in which vascular hyperpermeability plays a role in pathogenesis [22], which was accompanied by histological evidence of myocardial fibrosis and edema [23].

The integrity of myocardial vasculature and the overall contribution of cardiac dysfunction to the protracted shock associated with acute SCLS episodes are unknown. In the previously reported patient with histopathological evidence of myocardial edema, electrocardiogram (ECG) changes consistent with acute ischemia and elevations of cardiac troponin I were present transiently [9]. Although left heart catheterization revealed normal coronary arteries, echocardiography demonstrated increased thickness of both ventricular walls and globally reduced biventricular systolic function. By contrast, we found no ECG evidence of ischemia in our patient during the episode, and cardiac systolic function was reported to be normal by echocardiography. Thus, further studies will be needed to determine whether myocardial edema due to fluid leakage from coronary vessels represents a primary pathomechanism of SCLS or develops secondarily as a result of the multitude of metabolic and/or hematological derangements that typify the course of an acute SCLS attack.

For patients with acute SCLS flares, ECV mapping could provide a non-invasive quantitative assessment of the degree of myocardial edema at the time of the study. This information could serve as a marker for the diagnosis of SCLS together with the characteristic laboratory abnormalities (elevated hemoglobin, hypoalbuminemia) associated with an acute episode. Serial measurements of ECV could also serve as markers of treatment efficacy and/or disease progression. It is worth noting that approximately 65 % of the patients undergoing CMR in this study were receiving prophylactic treatment with either theophylline-based regimens or IVIG at the time of the procedure. As we noted, these therapies, in particular IVIG, have been shown to induce disease remission in many patients who receive them [6, 24]. It is possible that ECV values in the SCLS cohort were skewed by effective therapy. Given the rarity of this disorder and the inability to conduct large-scale clinical trials powered for hard clinical endpoints, ECV measurements could be used as a surrogate endpoint for studies of pharmacologic efficacy in the treatment of SCLS.

Unfortunately, the number of patients available for CMR immediately post-crisis was too small to allow a separate comparison with subjects with sporadic disease in remission or a determination of whether a correlation existed between ECV values and the time since the most recent episode. Our sample size was also too small to compare ECV values between treated and untreated patients,

## Conclusions

Patients with SCLS have a significantly higher myocardial ECV than age-matched controls. Elevations in ECV are most pronounced in patients with a recent SCLS flare or patients with a chronic form of SCLS. Myocardial ECV may serve as a diagnostic marker of SCLS and may aid in the evaluation of future treatments and prognosis in this rare disorder.
